# CD4^+^ T cell signature in long COVID: insights from an unvaccinated cohort

**DOI:** 10.3389/fimmu.2026.1823850

**Published:** 2026-06-29

**Authors:** Thiago Cerqueira-Silva, Benjamin Goodwin, Cíntia Araújo, Jessica J. Silva, Blenda de J. Pereira, Ícaro Bonyek Santos da Silva, Sara Nunes, Ananda Marinho, Ana Paula Barreto, Marcio Barreto, Marcelo Chalhoub, Juliana Ribeiro Caldas, Vishal Rao, Camila Coelho, Adolfo Rojas-Hidalgo, Vinicius Maracaja-Coutinho, Ricardo Khouri, Cristina R. Cardoso, Aldina Barral, Manoel Barral-Netto, Natalia Machado Tavares, Jennifer Dan, Viviane S. Boaventura

**Affiliations:** 1 Precision Medicine and Public Health, Gonçalo Moniz Institute, Fiocruz, Salvador, Bahia, Brazil; 2 Faculty of Epidemiology and Population Health, London School of Hygiene and Tropical Medicine, London, United Kingdom; 3 Instituto Nacional de Ciência e Tecnologia em Saude Digital (INCT- DigiSaude), Salvador, Brazil; 4 La Jolla Institute for Immunology (LJI), San Diego, CA, United States; 5 Federal University of Bahia, Salvador, Bahia, Brazil; 6 Hospital Especializado Octávio Mangabeira (HEOM), Salvador, Brazil; 7 Escola Bahiana de Medicina e Saúde Pública (EBMSP), Salvador, Brazil; 8 Department of Microbiology, Center for Vaccine Research and Pandemic Preparedness, and Precision Immunology Institute, Icahn School of Medicine at Mount Sinai, New York, NY, United States; 9 Albert Einstein Israelite Hospital, São Paulo, Brazil; 10 Unidad de Genómica Avanzada (UGA), Advanced Center for Chronic Diseases (ACCDiS), Facultad de Ciencias Químicas y Farmacéuticas, Universidad de Chile, Santiago, Chile; 11 Department of Microbiology, Immunology and Transplantation, Rega Institute for Medical Research, Laboratory of Clinical and Epidemiological Virology, Louven, Belgium; 12 Department of Clinical Analyses, Toxicology and Food Sciences, School of Pharmaceutical Sciences of Ribeirão Preto, University of São Paulo, Ribeirão Preto, Brazil; 13 Instituto Nacional de Ciência e Tecnologia, Instituto de Investigação em Imunologia (INCT - iii), São Paulo, Brazil

**Keywords:** ancestral, COVID-19, long covid, single cell RNA (scRNA), unvaccinated

## Abstract

**Objective:**

The pathogenesis of Long COVID (LC) remains poorly understood, with the analysis of immune responses often obscured by variables such as vaccination and reinfection. This study aimed to determine the primary immunological footprint of LC by examining a specific cohort that minimizes these confounding factors.

**Methods:**

We analyzed a Brazilian cohort of patients recruited between September 2020 and February 2021, during the first wave of the pandemic (Wuhan-Hu-1 variant), comprising mostly unvaccinated individuals. We assessed humoral responses to SARS-CoV-2 and latent viruses in 104 patients. Additionally, we investigated the immune signatures of CD4^+^ T cells in a subset of 6 LC and 4 recovered control (RC) unvaccinated patients, recruited 1 to 2 months after symptom onset, utilizing a combination of flow cytometry and single-cell RNA sequencing.

**Results:**

Systemic humoral responses to SARS-CoV-2 and reactivated latent viruses were comparable between the LC and RC groups, as were the overall distributions of CD4^+^ T cell subsets. However, single-cell RNA sequencing revealed distinct immune signatures. Antigen-responsive CD4^+^ T cells in LC patients demonstrated a signature of acute activation, characterized by the upregulation of genes such as *CD38* and interferon-stimulated genes, including *IFITM1*. Furthermore, gene set enrichment analysis indicated that LC patients exhibit increased expression of interferon-alpha/gamma signatures compared to RC. Analysis of the T cell receptor repertoire presented no evidence of clonal expansion.

**Discussion:**

Our findings imply that LC immunopathology is driven by a qualitative T cell dysfunction characterized by CD4^+^ non-proliferative activation more than one month after symptom onset. This pattern is consistent with persistent antigen stimuli, though the underlying mechanism and its therapeutic implications require further investigation.

## Introduction

Long COVID (LC), a multisystemic condition persisting beyond acute SARS-CoV-2 infection, represents a significant global health challenge affecting millions of people worldwide (1). Current evidence suggests that LC pathogenesis involves a complex interplay between viral persistence, chronic inflammation, autoimmunity, and reactivation of latent viruses, such as Epstein-Barr and herpesvirus (2, 3). Therefore, individuals with LC are anticipated to exhibit distinct cellular and humoral immune signatures compared to recovered controls (RC) (3–5). Exploring the adaptive immune response is essential to understanding immunity in LC. Perturbations in CD4^+^ T cell function, T-cell receptor (TCR) repertoire diversity, and sustained antibody responses to SARS-CoV-2 or reactivated pathogens may indicate underlying immune dysregulation (2, 5, 6).

CD4^+^ T cells play a central role in antiviral and humoral responses. However, their specific role in LC development remains incompletely characterized (3, 7). Recently, perturbations in CD4^+^ T cell function, including altered activation states and dysregulated inhibitory receptor expression, have been identified as playing a role in LC pathogenesis (5, 8). A proteomic study identified inflammatory phenotypes associated with LC, such as NF-κB and type 2 interferon signaling pathways (9). However, another study analyzing plasma from patients in Sweden and the United Kingdom found no significant differences, even though healthy controls exhibited higher neutralizing antibodies against SARS-CoV-2 (10). This study was conducted in 2022, after the rollout of the vaccine. A critical challenge in LC research has been distinguishing its unique immunological footprint from confounding factors such as COVID-19 vaccination and/or reinfection, which have complicated comparative studies years into the pandemic. Isolating LC-specific immune alterations remains challenging in cohorts exposed to vaccines or those with recurrent infections, both of which independently modulate adaptive immunity.

The heterogeneity of LC further complicates research efforts, with multiple potential mechanistic drivers and clinical manifestations requiring careful stratification of patient populations.^1,7^ To address these challenges, we evaluated CD4^+^ T cell activity in patients with LC compared to recovered patients (RC). We analyzed patients from Brazil’s first pandemic year (March 2020–March 2021), in which predominantly only ancestral (Wuhan-Hu-1) and Gamma SARS-CoV-2 variants were circulating (11, 12). These two variants had a negligible risk of reinfection and with reinfection being less likely within the first few months post infection (13), this reduced the influence of confounders in the immune signature due to vaccination or reinfection. This approach enables a more accurate assessment of the differences in immune signatures between LC and RC participants, providing valuable insights into the fundamental immunological mechanisms underlying LC pathogenesis.

## Methods

### Study design and participants

This cross-sectional study included 104 participants (79 with Long COVID [LC] and 25 recovered controls [RC]) selected from a previously described cohort (14). Individuals were recruited at the reference state Post-COVID Center in Salvador, Bahia, Brazil, between September 2020 and March 2021. Inclusion criteria specified at least one month since symptom onset of COVID-19 confirmed by RT-PCR, serology, or compatible computed tomography scan of the chest. Exclusion criteria included age under 18 years, presence of cognitive disorders, and pregnancy.

Participants underwent multidisciplinary evaluation at the outpatient clinic and were classified based on acute-phase disease severity as mild (no hospitalization), moderate (hospitalized but not requiring intensive care unit), or severe (requiring intensive care unit admission). Data were collected by trained physicians and nurses using standardized forms and managed via REDCap. Participants were classified as LC if they presented persistent symptoms for more than four weeks after disease onset, according to the CDC definition at the time of recruitment (2020–2021) (15).

To reduce confounding by age and sex, samples from the RC group were matched to the LC group by age (± 3 years) and sex at a 1:1 ratio, with replacement. To ensure representative LC symptom coverage, we performed cluster analysis evaluating 1,094 participants with complete symptom information using Gower distance and Partitioning Around Medoids method, resulting in 8 symptom clusters (**Supplementary Methods**). For single-cell analysis, a subset of 21 unvaccinated participants (16 LC, 5 RC) with available PBMC samples was selected after excluding 3 vaccinated individuals (**Supplementary Methods**). The study was approved by the institutional review boards of Bahia State University (UNEB, protocol no. 38281720.2.0000.0057) and Santo Antônio Hospital (OSID, protocol no. 33366030.5.0000.0047), and all participants provided written informed consent.

### Sample collection and processing

Whole blood was collected from participants in heparin or EDTA-coated tubes, with processing initiated within four hours of collection. PBMCs were isolated from heparin tubes using Ficoll gradient separation and cryopreserved in cell freezing medium (90% heat-inactivated fetal bovine serum with 10% DMSO) until further use.

### Humoral response assessment

Antibody responses against SARS-CoV-2 Spike RBD, Nucleocapsid, EBV EBNA1, and CMV pp65 were assessed via ELISA in all plasma samples (n=101), as previously described (16). Briefly, heat-inactivated plasma samples were serially diluted and incubated on 96-well plates coated with recombinant proteins. Negative control plasma was pooled from healthy donors unexposed to SARS-CoV-2, while positive control plasma from convalescent donors was used to normalize results across experiments. The limit of detection was defined as 1:3 (**Supplementary Methods**).

Neutralizing antibody activity was measured using a SARS-CoV-2 pseudovirus neutralization assay (16). Recombinant SARS-CoV-2-spike pseudotyped VSV-ΔG-GFP with the D614G mutation was incubated with serially diluted heat-inactivated plasma and added to confluent Vero cell monolayers. After 16 hours, cells were fixed and imaged to quantify infection. Neutralization titers (ID50) were calculated using the One-Site Fit Log IC50 model in GraphPad Prism 8.0.

### CD4+ T cell analysis

CD4^+^ T cells were evaluated using an activation-induced marker (AIM) assay, as previously described (17). Briefly, cryopreserved PBMCs were thawed, washed and cultured for 24 hours at 37 °C and 5% CO_2_ with SARS-CoV-2 MPs containing spike and non-spike epitopes (1μg/mL), DMSO (negative control), or staphylococcal enterotoxin B (1μg/mL, positive control). Cells were pre-incubated with anti-human CD40 blocking antibody (0.5μg/mL) for 15 minutes prior to stimulation.

Following stimulation, cells were stained with fluorochrome-conjugated antibodies against CD4, CD45RA, CCR7, CD27, OX40, 41BB, CD19, CD14, and CD16 (**Supplementary Methods**) and analyzed on a FACSAria IIu cell sorter (BD Biosciences). CD4^+^ T cells were classified into naïve (CCR7^+^CD45RA^+^), central memory (CCR7^+^CD45RA^−^), effector memory (CCR7^−^CD45RA^−^), and terminally differentiated effector memory (CCR7^−^CD45RA^+^) subsets. AIM^+^ cells were identified as OX40^+^41BB^+^. Three populations (naïve, total memory, and AIM^+^) were sorted for subsequent single-cell RNA sequencing, with viability confirmed before experiments.

### Single-cell RNA sequencing

For sorted cells, indexed V(D)J, 5’ Feature Barcode, and GEX libraries were prepared using the Chromium Next GEM Single Cell 5’ v2 Dual Index kit with Feature Barcoding (10x Genomics). Pools of cells of the same subtype from five to seven individuals were obtained following the Cell Multiplexing Oligo Labeling protocol. Libraries were sequenced on Illumina NovaSeq platform.

Initial data processing used CellRanger v7.1.0 (10x Genomics) (18) for demultiplexing and alignment to human reference genome GRCh38. Quality control excluded doublets identified by scDblFinder v1.16.0 and cells with <200 or >6,000 detected genes or >10% mitochondrial RNA content. Data analysis was performed using Seurat v5.0.3 (**Supplementary Methods**).

After log-normalization (10,000 reads), dimensionality reduction was performed using PCA on the 2,000 most variable genes, excluding *MALAT1*, ribosomal, and mitochondrial genes. The first 22 principal components (accounting for 80% of variance) were selected. Batch effects were corrected using Harmony v1.2.0, and data were visualized using UMAP with default Seurat implementation. Clustering used FindNeighbors (k=10) and FindClusters functions (**Supplementary Methods**).

Cell type annotation employed SingleR v2.4.0 with four reference datasets: Blueprint Encode Data, Database Immune Cell Expression Data, Human Primary Cell Atlas Data, and Monaco Immune Data. Annotations were consolidated into four categories: naïve, memory, regulatory (Tregs), and activated T cells. T cell receptor (TCR) sequences were integrated with transcriptomic profiles by merging clonotype and AIRR metadata files. TCR β-chain sequences were clustered using CD-HIT v4.8.1 at 85% sequence identity and matched against the VDJdb database for antigen specificity annotation, including SARS-CoV-2, CMV, EBV, and autoantigens (**Supplementary Methods**).

### Statistical analysis

Differences in antibody titers and neutralization titers between the LC and RC groups were evaluated using the Wilcoxon rank sum test. Categorical variables were compared using Pearson’s chi-squared test. The difference in CD4^+^ T cell subset proportions between groups was evaluated using compositional data transformation (isometric log-ratio) followed by Permutational Multivariate Analysis of Variance (PERMANOVA), with 9999 permutations.

Differential gene expression analysis employed a pseudobulk approach, aggregating single-cell data by summing expression counts for each gene across cells of the same cell type and individual, using a modified version of the Libra R package. Individuals with fewer than 10 cells per cell type comparison were excluded to minimize bias from low cell representation. DESeq2 with Wald test was used for statistical testing, with significant genes identified at false discovery rate (FDR)-adjusted p-value <0.1.

Gene set enrichment analysis (GSEA) was performed using fgsea v1.30.0 in pre-ranked mode. Genes were scored using the formula: score = −log_10_(p-value) × sign(log_2_FC). We used the MSigDB v2024.1.Hs Hallmark gene sets (19), a curated collection of 50 well-defined biological processes. Genes not expressed in any sample were removed from gene sets to reduce bias (20). Pathway enrichment was defined as FDR <0.05. Similarities in gene dysregulation patterns between groups were evaluated by the Normalized Enrichment Score. All analyses were performed in R v4.3.3.

## Results

This study cohort consisted of 79 individuals with LC and 25 recovered controls (RC), mostly unvaccinated at the time of infection (101 - 97.1%) and with no history of reinfection following their primary infection, during the first year of the pandemic (**Table 1**, **Supplementary Figure 2**). A sub-cohort of 16 LC and 5 RC participants, all of whom were unvaccinated at the time of sample collection, underwent single-cell CD4^+^ analysis; among them, only 10 (6 LC and 4 RC) were analyzed after quality control. There were no significant demographic or comorbidity differences between the groups (**Table 1**, **Supplementary Figure 3**).

**Table 1:** Characteristics of the participants.

Characteristic	All participants		p-value	T-cell study		p-value
	Recovered, N = 25	Long-COVID, N = 79		Recovered, N = 4	Long-COVID, N = 6	
Age - years, median (IQR)	55 (49 - 63)	57 (52 - 64)	>0.9	57 (52 - 59)	56 (54 - 63)	>0.9
Sex - female	10 (40)	42 (53)	0.3	1 (25)	3 (50)	>0.9
BMI (kg/m2), median (IQR)	28.5 (25.7 - 31.6)	28.2 (26.0 - 31.0)	0.7	26.0 (24.4, 32.8)	27.5 (24.4, 28.6)	>0.9
Missing	0	2				
Severity			0.8			0.5
Mild	7 (28)	24 (30)		0 (0)	0 (0)	
Moderate	9 (36)	32 (41)		2 (50)	5 (83)	
Severe	9 (36)	23 (29)		2 (50)	1 (17)	
Heart disease	2 (8.0)	11 (14)	0.7	1 (25)	1 (17)	>0.9
Hypertension	16 (64)	39 (49)	0.2	1 (25)	3 (50)	0.6
Diabetes Mellitus	6 (24)	19 (24)	>0.9	1 (25)	1 (17)	>0.9
Chronic obstructive pulmonary disease	1 (4.0)	3 (3.8)	>0.9	0 (0)	0 (0)	>0.9
Smoking			0.2			0.7
Never	14 (58)	58 (73)		1 (25)	3 (50)	
Past	10 (42)	18 (23)		3 (75)	2 (33)	
Current	0 (0)	3 (3.8)		0 (0)	1 (17)	
Missing	1	0				
Alcohol consumption			0.4			0.5
Never	12 (50)	47 (61)		3 (75)	2 (33)	
Current occasional drinker	11 (46)	29 (38)		1 (25)	4(67)	
Current daily drinker	1 (4.2)	1 (1.3)		0 (0)	0 (0)	
Missing	1	2		0	0	
Months post symptom onset, median (IQR)	2.60 (1.60 - 4.50)	2.20 (1.60 - 3.85)	0.5	2.1 (1.55 - 4.9)	1.40 (1.40 – 1.40)	0.04
First dose of COVID-19 vaccine before infection	0 (0)	3 (3.8)	>0.9	0 (0)	0(0)	>0.9
First dose of COVID-19 vaccine before sample collection	2(8)	6 (7.6)	>0.9	0 (0)	0(0)	>0.9

The p-values are from the Wilcoxon rank sum test or Pearson’s Chi-squared test. Numbers are N(%) or otherwise specified.

**Figure 1:**
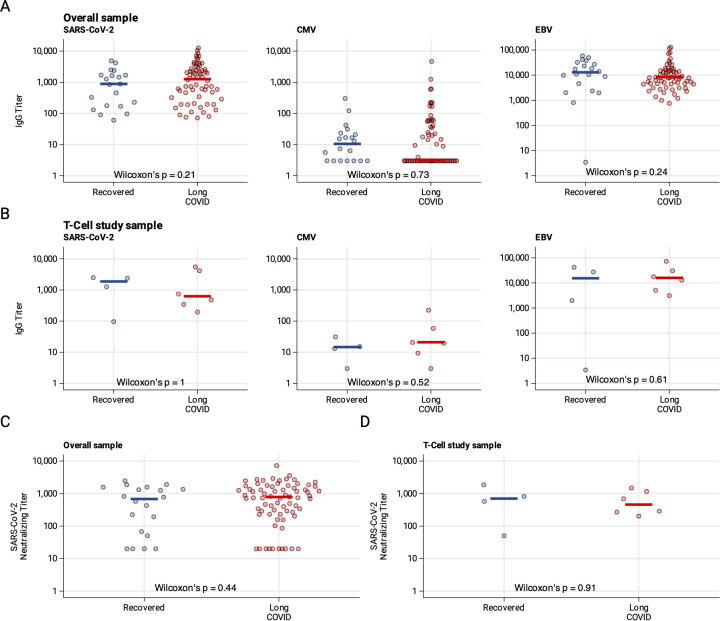
Humoral response to SARS-CoV-2, CMV, and EBV in recovered and long COVID individuals. Overall sample **(A, C)** and subset from the T-cell study **(B, D)**. **(A)** Overall IgG titers against SARS-CoV-2 RBD, CMV, and EBV antigens; **(B)** IgG titers in the subset of participants analyzed in the T cell single-cell RNAseq; **(C)** IgG neutralizing activity against SARS-CoV-2; **(D)** IgG neutralizing activity against SARS-CoV-2 in the subset of participants analyzed in the T cell single-cell RNAseq. The horizontal line represents the median value in each group. EBV, Epstein–Barr virus; CMV, Cytomegalovirus.

First, we evaluated key components of virus-specific immunity. Analysis of humoral responses revealed no statistically significant differences in anti-Spike IgG antibody titers or SARS-CoV-2 neutralizing antibody activity between the LC and RC groups. Furthermore, serological analysis showed no evidence of differential reactivation of latent viruses, with comparable IgG titers for both Epstein–Barr virus (EBV) and Cytomegalovirus (CMV) (**Figure 1**).

**Figure 2:**
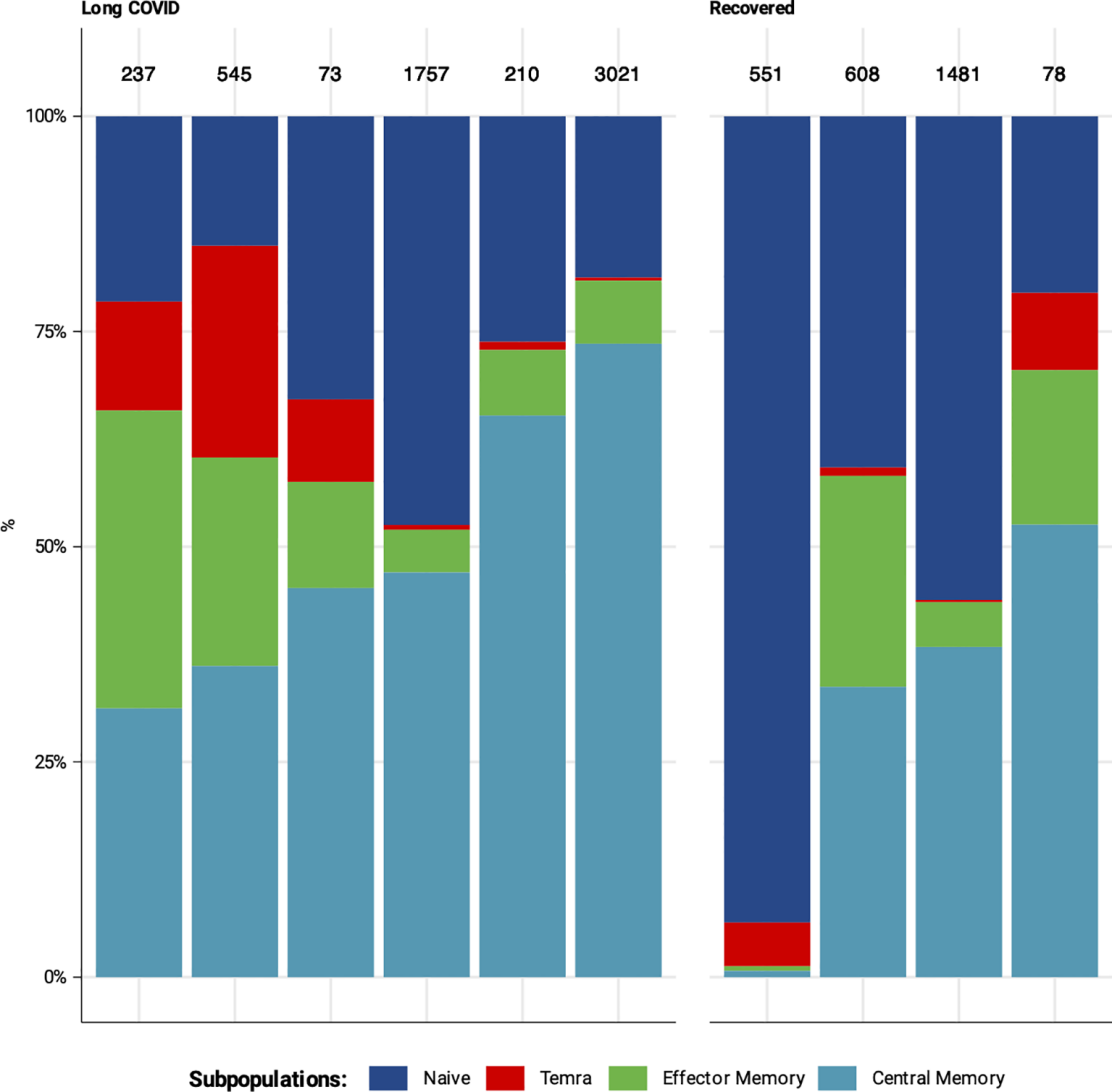
Distribution of AIM^+^ CD4^+^ T cell subsets in individuals with long COVID and recovered controls analyzed in the single-cell RNAseq. Flow cytometry was used to analyze CD4^+^T cells after excluding non-T cells (CD19^+^, CD14^+^, and CD16^+^). CD4^+^T cells were further classified into naïve (T_N_, CD45RA^+^ CCR7^+^), central memory (T_CM_, CD45RA- CCR7+), effector memory (T_EM_, CD45RA^-^ CCR7^-^), and terminally differentiated effector memory (T_EMRA_, CD45RA^+^ CCR7^-^) subsets. Numbers in each bar represent the total number of AIM^+^ cells.

To investigate the role of *CD4^+^* T cells in LC pathogenesis, we classified *CD4^+^* T cells into three subsets using flow cytometry: total memory (*CCR7^+^CD45RA^-^*, *CCR7^-^CD45RA^-^, CCR7^-^CD45RA^+^*), naïve (*CCR7^+^CD45RA^+^*), and activation-induced markers (AIM^+^) (*OX40^+^41BB^+^*). Total memory and naïve groups were capped at a maximum of 2900 cells. The number of AIM^+^ cells per sample and the distribution of the subpopulations are shown in **Figure 2**. Naïve and central memory were the most prevalent subpopulations; the distributions of naïve, central memory, effector memory, and terminally differentiated effector memory were similar between LC and RC participants, as indicated by a PERMANOVA p-value of 0.53 (**Figure 2**).

**Figure 3:**
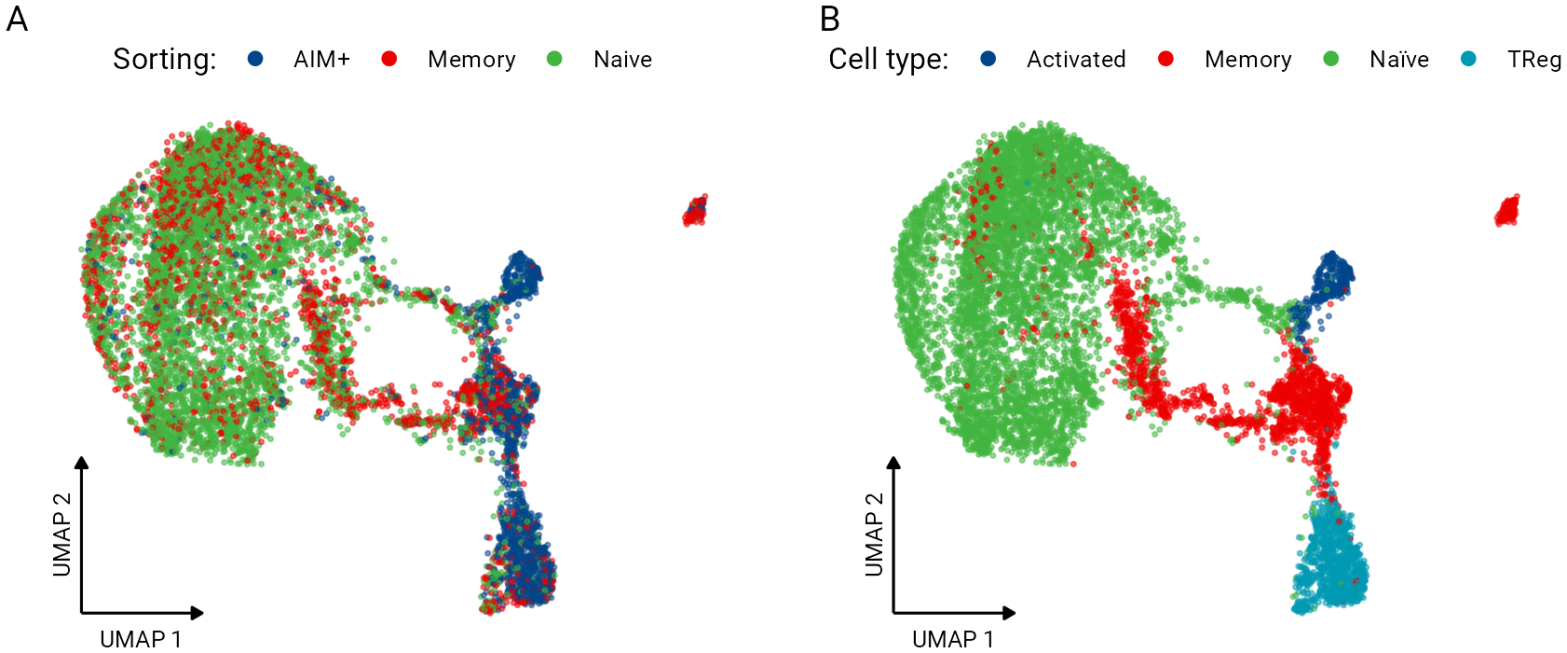
Single-cell characterization and reclassification of CD4^+^ T cell subsets from all individuals (long COVID and recovered controls). CD4^+^ T cells initially sorted by flow cytometry into naïve, memory, and activation-induced marker-positive (AIM^+^) subsets underwent single-cell RNA sequencing (scRNA-seq). The cells from each sorted subset were further annotated based on transcriptional profiles and visualized using Uniform Manifold Approximation and Projection (UMAP). **(A)** The left panel shows a UMAP visualization of the sorted populations (naïve, memory, AIM^+^). **(B)** The right panel presents the reclassification of cells into four transcriptionally distinct subsets: naïve, activated, memory, and regulatory T cells (Tregs).

To further characterize *CD4^+^* T cells, we performed single-cell RNA sequencing (scRNA-seq) of the groups sorted in the flow cytometry (naïve, total memory, and AIM^+^ cells). We further classified the T cells within each sorting based on the expression profiles of marker genes from the scRNA-seq: naïve, activated, memory, and regulatory (TReg) (**Figure 3**). Most cells (87%) sorted as naïve in the flow cytometry were annotated as naïve based on their gene expression profiles, reinforcing the sorting accuracy and overall data quality. Within the AIM^+^ sorted population, the proportions of subsets by gene expression were Tregs (39%), activated (17%), memory (31%), and naïve (12%) T cells, indicating that AIM^+^ sorting successfully enriched antigen-responsive cells (**Supplementary Figure 4**).

**Figure 4:**
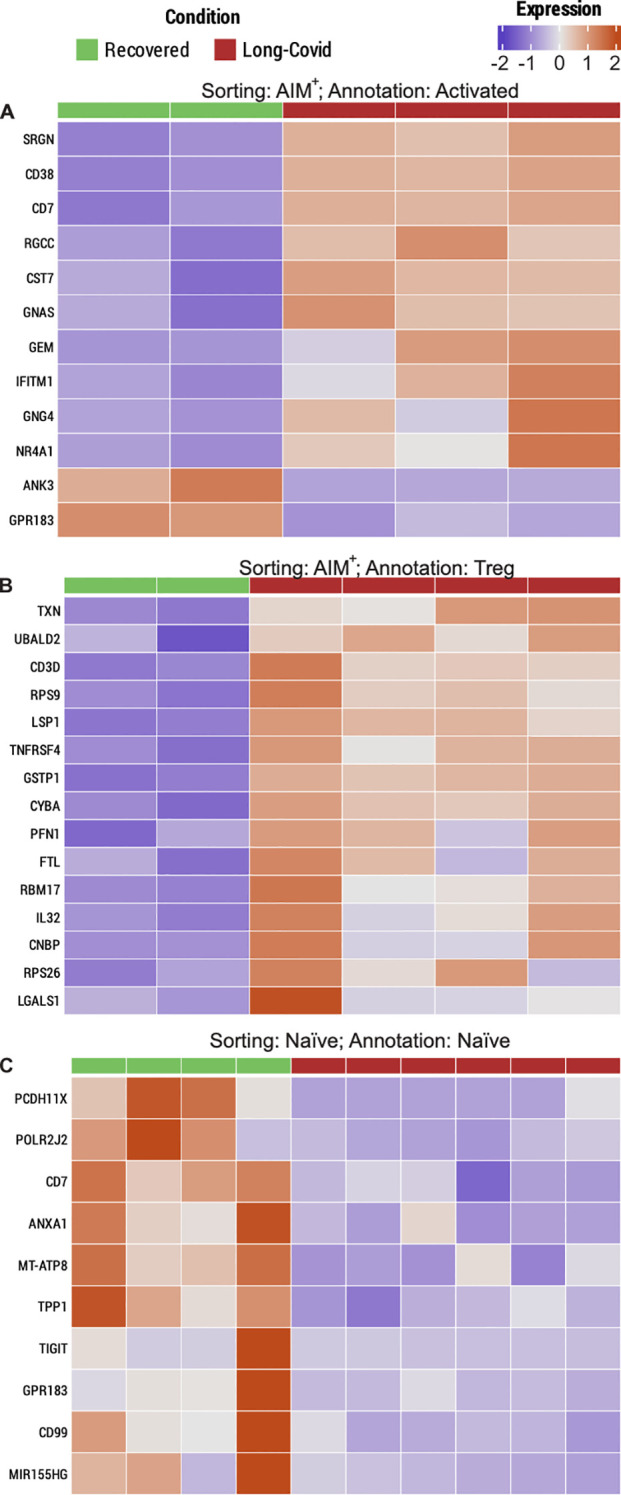
Differential gene expression analysis across CD4^+^ T cell subsets in long COVID and recovered controls. Heatmaps display genes that are differentially expressed between LC and recovered individuals, categorized by sorting strategy and cell annotation based on single-cell RNA sequencing. **(A)** Cells sorted as AIM^+^ and annotated as activated; **(B)** Cells sorted as AIM^+^ and annotated as regulatory T cells (Treg); **(C)** Cells sorted as naïve and annotated as naïve. Color gradients represent relative gene expression levels (upregulated in red, downregulated in violet). Each column represents the aggregated expression profile of a single participant.

To ensure rigorous quality control in the bioinformatics analysis comparing LC vs. RC, individuals with fewer than 10 cells detected in any subset of sorting and gene expression were excluded. This filtering step minimized bias from low cell representation and enhanced the reliability of downstream analyses. We compared the transcriptional profiles of cells classified by gene expression annotation within each flow-cytometry sorting gate. We used the notation *Gene-expression__sorting_*, to refer to groups within each sorting. We identified 12 differentially expressed genes (DEGs) in *activated__AIM_^+^* cells (2 RC and 3 LC patients), with 10 upregulated genes in LC patients. Among these genes, *IFITM1* and *CD38* were notably overexpressed in the LC group (**Figure 4A**, **Supplementary Table 1**). We also identified 15 DEGs in the *Treg__AIM_^+^* cells (2 RC and 4 LC patients), which were upregulated in the LC group (**Figure 4B**, **Supplementary Table 2**). Lastly, in the *Naïve__Naïve_*,(4 RC and 6 LC patients) we identified 10 DEGs in this group, all of which were downregulated in the LC group (**Figure 4C**, **Supplementary Table 3**). The remaining groups (*Memory__Memory_* and *Naïve__Memory_*) did not exhibit any DEGs.

**Figure 5:**
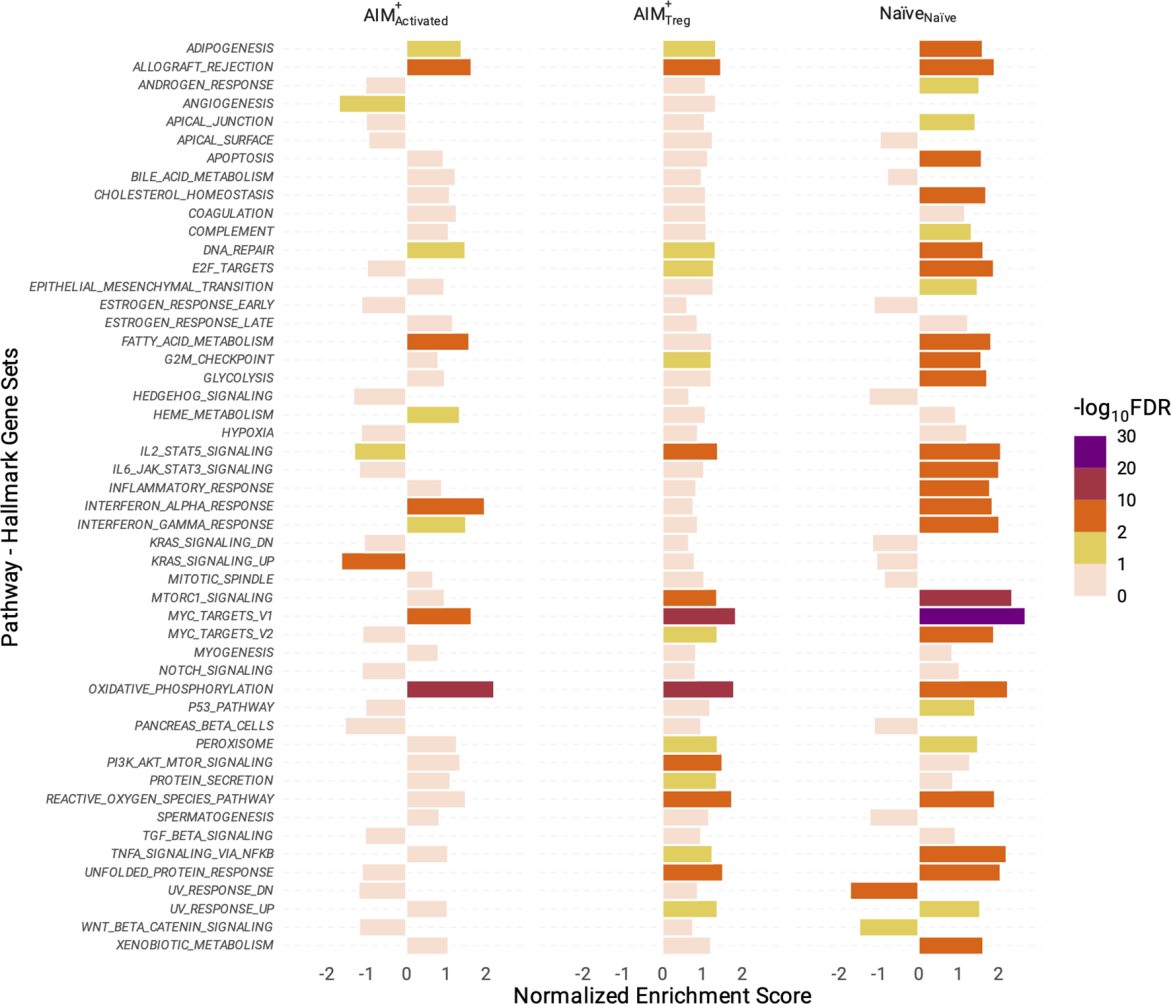
Gene set enrichment analysis (GSEA) of CD4^+^ T cell sorted as activated induced marker by functional annotation subsets. GSEA plots of hallmark pathways comparing Long Covid to Recovered patients across three distinct subsets: Activated, regulatory T cells (Treg), and naïve. The normalized enrichment scores reflect the magnitude and direction of enrichment in LC individuals. FDR = False discovery rate.

In the gene set enrichment analysis (GSEA), *activated__AIM+_* CD4^+^ cells were enriched for interferon alpha and gamma responses in LC (**Figure 5**), whereas *Tregs__AIM+_* were enriched for IL-2 signaling responses (**Figure 5**). *Naïve__Naïve_* cells from LC showed increased interferon alpha and gamma responses, *IL-2*, *IL-6*, and *TNF-α* signaling responses, and overall inflammatory responses compared to RC (**Figure 5**, **Supplementary Figure 5**).

**Figure 6:**
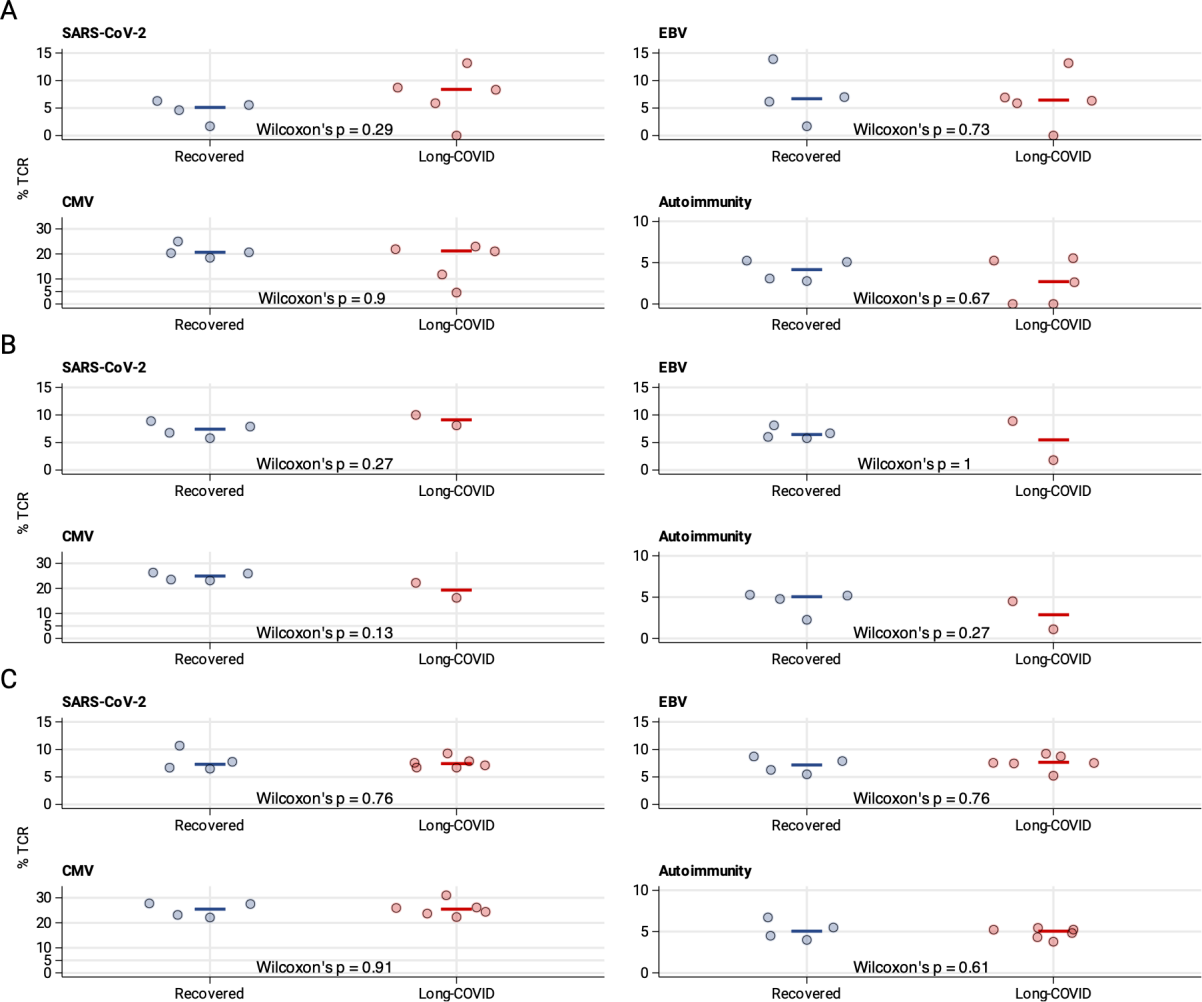
T-cell receptor (TCR) repertoire analysis across CD4^+^ T cell subsets in long COVID and recovered participants. TCR sequences derived from single-cell RNA sequencing were analyzed and cross-referenced with public databases of known antigen-specific TCRs (SARS-CoV-2, EBV, CMV, and autoimmunity). The figure depicts the percentage of antigen-specific TCR within three subsets sorted by flow cytometry: **(A)** AIM^+^, **(B)** Total Memory, and **(C)** Naïve CD4^+^ T-cells.

To determine whether the observed transcriptomic activation was associated with the expansion of SARS-CoV-2-specific T cells, we analyzed the TCR sequences obtained from the scRNA-seq data. We identified TCR clonotypes specific to SARS-CoV-2 using public databases. We did not find any significant differences in the overall clonal diversity of the TCR repertoire between the LC and RC groups (**Figure 6**). We also did not find evidence of clonal expansion among the sorted *CD4^+^* T cells (activated, total memory, and naïve). (**Supplementary Figure 6**).

## Discussion

Our study provides important insights into the immunological mechanisms underlying LC, particularly focusing on CD4+ T cell dysregulation in an unvaccinated cohort with minimal reinfection risk. This unique cohort allowed us to isolate LC-specific immune alterations without the confounding effects of vaccination or reinfection. We found that while individuals with LC do not differ from those with RC in the quantity and quality of their humoral response or the overall distribution of CD4^+^ T-cell subsets, their antigen-responsive T-cells exhibit a distinct immune signature. Indeed, we observe a strong interferon signature more than 1 month after symptom onset in individuals with LC.

The genes of AIM^+^ CD4^+^ T-cells exhibited differential expression patterns between LC and recovered individuals. Notably, LC patients showed upregulation of acute activation markers, such as *CD7* and *CD38*, along with the interferon-stimulated gene *IFITM1*, indicating active immune stimulation. Although *IFITM1* is broadly associated with antiviral activity, *in vitro* evidence suggests it may facilitate SARS-CoV-2 entry during acute infection (21), though its relevance in the post-acute setting remains unclear. *CD38* is a classic marker of activation in viral infections (22, 23). The role of *CD7* in CD4^+^ T cells is not well known. It has been mostly studied in the context of tumor biology, where CD7^-^CD4^+^ T cells have been observed in T-cell lymphoma (24). A 2024 study showed that CD7^+^ CD4^+^ T cells are associated with cytolytic activity in autoimmunity (25). Our findings of active immune response were further substantiated by gene set enrichment analysis, which revealed significant enrichment of interferon-gamma and interferon-alpha response pathways in activated cells from the LC group. The SARS-CoV-2-specific CD8^+^ T cells from LC patients showed increased expression of co-inhibitory receptors, suggesting a state of T cell dysfunction that may contribute to persistent symptoms (10). In contrast, Yin et al. (5) examined a similarly unvaccinated, low-reinfection-risk population eight months post-disease and did not observe differences in activation markers, such as *CD38*, on CD4^+^ or CD8^+^ T cells when comparing the LC group to the recovered group. Instead, their study showed a differential expression of exhaustion markers, including *PD-1* and *CTLA-4*. Considering the timing of patient recruitment between our study (1–2 months post disease) and Yin et al. (8 months post disease). One possible interpretation, when considering these two independent cohorts, is that immune activation in the early months post-infection may precede a transition toward exhaustion at later timepoints. However, this comparison is limited by differences in cohort characteristics, including disease severity and geographic setting, and would require longitudinal confirmation within a single cohort.

Despite the findings of immune activation in SARS-CoV-2-specific CD4^+^ T-cells, our analysis did not reveal a difference in response towards SARS-CoV-2 antigen. We found no evidence of clonal expansion of T-cell receptors in any CD4^+^ cell population. The absence of detectable differences in viral-specific T-cell frequencies between LC and RC could partially be attributed to the relatively small sample size, which limits statistical power, and the possible lack of matches in the VDJdb database. The combination of immune activation markers without corresponding clonal expansion is consistent with a model in which a low-level antigen stimulus sustains inflammatory signaling without eliciting a proliferative T-cell response. One candidate source for such stimulation is a tissue-resident viral antigen reservoir, which has been documented in other studies (2, 26, 27), although our data cannot directly test this mechanism. Whether this state precedes T-cell exhaustion, as observed in other post-viral conditions, remains to be determined.

Lastly, we have also evaluated alternative hypotheses of LC, such as the reactivation of a latent virus (2, 28, 29). Our analysis showed no significant difference in EBV-specific or CMV-specific IgG antibody titers between the LC and RC groups. The frequency of TCR against those viruses was similar in both groups. These results are similar to those of Williams et al. (30), who did not find a difference in the immune response of CD4^+^/CD8^+^ T-cells towards SARS-CoV-2, EBV, and CMV comparing LC and RC patients.

The primary strength of our study lies in the use of a cohort of mostly unvaccinated, non-reinfected individuals from the first wave of the pandemic, which provides an invaluable baseline for understanding the primary immunopathology of LC. We also provided a detailed clinical characterization of the individuals. We also combined flow cytometry and scRNA-seq on unvaccinated individuals to obtain complementary insights. Flow cytometry allowed us to quantify a predefined set of surface proteins, while scRNA-seq provides a broad, comprehensive transcriptomic profile at single-cell resolution (31). A previous study comparing human peripheral blood mononuclear cells found that the correlation between protein expression measured by cytometry and mRNA levels from scRNA-seq was relatively weak, with r-squared values ranging from 0.47 to 0.66 (32, 33). Therefore, since each technique targets different cellular components and relies on different markers to define subpopulations, their annotations do not always completely overlap. However, the benefit of applying scRNA-seq to a pre-sorted cell population is that its high resolution can uncover substantial heterogeneity within populations that were previously identified and isolated by flow cytometry. In this way, flow cytometric sorting serves as a useful pre-filter, providing an initial standardization of cell populations before transcriptomic analysis.

However, the study has limitations. First, the small sample size for the scRNA-seq precluded the assessment of the immune expression by different symptom clusters, as previous studies have shown that LC is not a homogenous condition, and it is likely to differ in immunological patterns (34, 35). The limited sample size also reduced the statistical power to detect differences and the generalizability of our findings. We initially planned to analyze samples from 9 RC and 27 LC participants, but due to technical problems with experiments and a limited number of AIM^+^ cells sorted, only 6 LC and 4 RC participants presented adequate numbers of cells for single-cell RNASeq analysis. Similarly, we used an FDR threshold of 0.1 for differential expression to account for the possible similarity in immune profiles between LC and RC. This more permissive threshold allowed us to detect subtle but potentially biologically relevant changes, at the cost of an increased risk of false positives among the identified genes. Second, analysis regarding TCR repertoire relied on the VDJdb, a repository containing high-quality, validated TCR sequences, however it can show low sensitivity to uncommon epitopes (36). Third, this cross-sectional study is a single snapshot in time; longitudinal follow-up is essential to understand the trajectory of this T-cell immune pattern. Fourth, our findings are based primarily on transcriptomic analysis, and the lack of available cell samples precluded functional validation of key pathways at the protein level. Finally, our analysis of peripheral blood may not fully capture the immunological events occurring within tissue sites of potential viral persistence.

In conclusion, by studying an unvaccinated cohort, we provide evidence of transcriptomic alterations in the CD4^+^ T-cell compartment in LC, characterized by immune activation signatures in antigen-responsive cells that are uncoupled from both clonal expansion and the systemic humoral response. These findings, observed in a small cohort, warrant validation in larger longitudinal studies.

## Data Availability

Sequence data that support the findings of this study have been deposited in the NCBI GEO database with the primary accession code GSE334857.

## Supplementary material

Supplementary Table 1 Log2 Fold changes and p-values of differentially expressed genes in the comparison activated_AIM+.

Supplementary Table 2 Log2 Fold changes and p-values of differentially expressed genes in the comparison Treg_AIM+.

Supplementary Table 3 Log2 Fold changes and p-values of differentially expressed genes in the comparison Naïve_Naïve.

Supplementary Figure 1 Dimensionality reduction through t-Distributed Stochastic Neighbor Embedding (t-SNE). Evaluating the presence of cough, fatigue, dyspnea, pain symptoms (headache, thoracic and muscle pain), and changes in taste and smell.

Supplementary Figure 2 Number of participants with each combination of symptoms and related cluster.

Supplementary Figure 3 Number of participants with each combination of symptoms and related cluster for the T-cell study.

Supplementary Figure 4 Percentage of cells by gene expression profile (scRNA-seq) within each sorting (flow cytometry).

Supplementary Figure 5 GSEA by cell sorting. **(A)** AIM^+^, **(B)** Memory, **(C)** Naïve6.

Supplementary Figure 6 TCR Expansion overall and by cell sorting. **(A)** Overall, **(B)** AIM^+^, **(C)** Memory, **(D)** Naive.
