# Supplementary material for: CD4+ T cell signature in long COVID: insights from an unvaccinated cohort
Source: Front Immunol. 2026 Jun 29;17:1823850. doi: 10.3389/fimmu.2026.1823850 (PMC13357944; doi:10.3389/fimmu.2026.1823850)
Supplement: Supplementary Table 1 — Log2 Fold changes and p-values of differentially expressed genes in the comparison activated_AIM+. [file Table1.docx]

Table of Contents

[Supplementary Methods 2](#_Toc228805126)

[Supplementary Table 1. Log_2_ Fold changes and p-values of differential expressed genes in the comparison activated__AIM+_ 12](#_Toc228805127)

[Supplementary Table 2. Log_2_ Fold changes and p-values of differential expressed genes in the comparison Treg__AIM+_ 12](#_Toc228805128)

[Supplementary Table 3. Log_2_ Fold changes and p-values of differential expressed genes in the comparison Naïve__Naïve_ 12](#_Toc228805129)

[Supplementary Figure 1: Dimensionality reduction through t-Distributed Stochastic Neighbor Embedding (t-SNE). Evaluating the presence of cough, fatigue, dyspnea, pain symptoms (headache, thoracic and muscle pain), and changes in taste and smell. 13](#_Toc228805130)

[Supplementary Figure 2: Number of participants with each combination of symptoms and related cluster. 14](#_Toc228805131)

[Supplementary Figure 3: Number of participants with each combination of symptoms and related cluster for the T-cell study. 15](#_Toc228805132)

[Supplementary Figure 4: Percentage of cells by gene expression profile (scRNA-seq) within each sorting (flow cytometry) 16](#_Toc228805133)

[Supplementary Figure 5: GSEA by cell sorting. A. AIM+, B. Memory, C. Naïve 17](#_Toc228805134)

[Supplementary Figure 6: TCR Expansion overall and by cell sorting. A. Overall, B. AIM+, C. Memory, D. Naïve 19](#_Toc228805135)

[Supplementary References 19](#_Toc228805136)

# Supplementary Methods

**Human subjects**

This cross-sectional study included 104 participants (79 with LC and 25 RC), who were selected from a cohort previously described.*(1)* Briefly, individuals were recruited at the reference state Post-COVID Center in Salvador, Bahia, Brazil, between September 2020 and March 2021. Inclusion criteria specified at least one month since symptom onset of COVID-19 confirmed by RT-PCR, serology, or compatible computed tomography scan of the chest. The exclusion criteria were: age under 18 years, presence of cognitive disorders, and pregnancy. In the outpatient clinic, individuals underwent multidisciplinary evaluation. They were classified based on acute-phase disease severity as mild (no hospitalization), moderate (hospitalized but not requiring an intensive care unit), or severe (requiring an intensive care unit). Data were collected by trained physicians and nurses using standardized forms and managed via REDCap. Sociodemographic and clinical data included respiratory, neurological, pain, and constitutional symptoms, as well as anthropometric parameters, oxygen saturation, comorbidities, and lifestyle habits. Individuals were classified as Long COVID if they presented persistent symptoms for more than four weeks after disease onset, according to the CDC definition at the time of recruitment (2020-2021).*(2)*

The 104 participants were selected by matching samples from the RC group to the LC group. We first defined symptom clusters for Long COVID participants (see Cluster Analysis below), then to reduce confounding by age and sex in the LC and RC groups, we selected samples from participants matched by age (±3 years) and sex. Participants from different clusters (LC group) were matched to the RC group at a 1:1 ratio. The samples from the RC group could be matched with samples from various symptom clusters, i.e., matching with replacement.

**Ethics**

The study was approved by the institutional review boards of Bahia State University - UNEB (protocol no. 38281720.2.0000.0057) and Santo Antonio Hospital - OSID (protocol no. 33366030.5.0000.0047), and all participants provided written informed consent.

**Cluster analysis for participant selection**

To ensure a representative range of LC symptoms, we defined symptom clusters by evaluating 1094 participants with complete information on symptoms (cough, fatigue, dyspnea, pain symptoms, change of smell/taste, and other symptoms). The distance between each participant was calculated using the Gower distance,*(3)* and the cluster analysis was conducted using the Partitioning around medoids method. We evaluated the range of clusters from 2 to 40, and the final number of clusters (30) was defined by assessing the silhouette width.*(4)* Further reclassified into 8 clusters: 1) only pain symptoms (thoracic pain, headache, myalgia), 2) only change in smell and/or taste, 3) only fatigue, 4) only cough, 5) only dyspnea, 6) pain, change in smell/taste, fatigue, cough, dyspnea), 7) mixed (multiple group of symptoms), and 8) only other symptoms (such as memory loss, insomnia, hair loss, loss of appetite, dysphagia, dysphonia, etc.). (Supplementary Figure 1)

**Sample collection and processing**

Whole blood was isolated from human study participants and collected in heparin or EDTA-coated tubes. Processing began within four hours after blood collection. PBMCs were obtained from heparin tubes using Ficoll gradient and cryopreserved in cell freezing medium consisting of 90% heat-inactivated fetal bovine serum with 10% DMSO until further use in experiments.

**Activation-induced marker assay (AIM)**

The activation-induced marker assay has been previously described in detail.*(5)* Briefly, frozen PBMCs were thawed, washed, and cultured for 24 hours at 37°C and 5% CO_2_ in the presence of SARS-CoV-2 MPs (containing spike (S) and non-spike epitopes (CD4-RE) at 1μg/mL), negative control DMSO (adding an equimolar amount of DMSO), or positive control SEB (staphylococcal enterotoxin B at 1μg/mL) in 96-well U-bottom plates. PBMCs were plated at 0.5-1.0x10^6^ cells per well. Prior to the addition of stimulation treatment, cells were incubated with 0.5μg/mL anti-human CD40 blocking antibody for 15 minutes at 37°C and 5% CO_2_. After the 24-hour incubation, 96 well plates were centrifuged, and PBMCs were washed with 5% FBS in PBS solution.

**Flow cytometry and cell sorting**

Cells were stained with following antibodies (Fluorochrome- Clone -Manufacturer) diluted 1:50: CD45RA (BV570 - HI100 - Biolegend cat#304133), 41BB (BV650 - 4B4-1 - Biolegend cat#3309828), CD4 (BV785 - OKT4 - Biolegend cat#3317442), OX40 (FITC - Ber-ACT35 - Biolegend cat#3350006), CD19 (PE-Cy7 - H1B19 - Invitrogen cat#25-0199-42), CD27 (PerCP-eFluor710 - 0323 - Invitrogen cat#46-0279-42), CCR7 (APC -G043H7 - Biolegend cat#353214); CD14 **(**APC-eFluor780 - eBio CB16 - Invitrogen cat# 47-0168-42), and CD16 ( APC-eFluor780- eBio CB16- Invitrogen -cat#47-0168-42).

All cells were analyzed and sorted on a FACSAria IIu cell sorter (BD Biosciences). CD4+T cells with total Memory (CCR7+CD45RA-, CCR7-CD45RA-, CCR7-CD45RA+), Naive (CCR7+CD45RA+), and AIM+ (OX40+41BB+) markers were sorted, and viability was analyzed before the scRNAseq experiment. The difference in the proportions of each cell type by group was evaluated using compositional data transformation (isometric log-ratio) and multivariate analysis of variance (MANOVA).

**Single-cell RNA sequencing**

Among the matched patients, 24 (RC=5 and LC=19) had PBMC collected samples. We excluded 3 patients (LC=3) who had been vaccinated before sample collection, resulting in individuals (n=21; n=5 RC and n=16 LC). For sorted cells, indexed V(D)J, 5’ Feature Barcode, and GEX libraries were prepared using the Chromium Next GEM Single Cell 5’ v2 Dual Index with Feature Barcoding Kit (10x Genomics). Libraries were pooled and sequenced on a NovaSeq Sequencer (Illumina).

Pools of cells of the same subtype, from five to seven individuals, were obtained following the Cell Multiplexing Oligo Labeling for Single Cell RNA Sequencing Protocol (10x Genomics). Size distribution and library concentration were determined using a Bioanalyzer High Sensitivity chip (Agilent Technologies). Sequencing was carried out on the Illumina NovaSeq platform.

**Initial data processing and quality control.**

The initial transcriptomic data were processed using CellRanger v7.1.0 (10x Genomics),*(6)* following the manufacturer’s guidelines for demultiplexing pooled libraries with hashtags. Reads were aligned to the human reference genome GRCh38, with FASTA and index files obtained from the 10x Genomics website. The resulting gene-count matrices were analyzed with the Seurat R package v5.0.3 (Satija et al., 2015; Butler et al., 2018) in R v4.3.3, setting a random seed of 123 to ensure reproducibility. Doublets were identified using scDblFinder v1.16.0, and cells flagged in the remove.doublet file were excluded from the dataset with the Seurat subset() function. Additional quality-control filters were applied to remove cells with fewer than 200 detected genes, more than 6,000 detected genes, or >10% mitochondrial RNA content.

To integrate T cell receptor (TCR) sequencing data with transcriptomic profiles, clonotype and AIRR metadata files from TCR-seq were merged using clonotype identifiers, generating a data frame with clonotype frequency, relative abundance, and *CDR3* sequences. *CDR3* chain composition was analyzed to determine the number and type of chains per cell, allowing identification of potential doublets through the detection of multiple TRA or TRB chains. The annotated TCR data were incorporated into the corresponding Seurat object, enriching it with immunological metadata and categorizing samples by condition and immune data availability. Finally, cells were filtered based on immune-related criteria, including the presence of TRA or TRB sequences, detection of TCR doublet patterns, and expression of *CD8A* and *CD8B*.

**Data normalization, dimensionality reduction, and clustering.**

Prior to the clustering and data visualization steps, data were normalized using the NormalizeData function from Seurat, considering default settings, resulting in a log-transformed transcript output per 10,000 reads. The normalized gene/barcode matrix was used to perform dimensionality reduction. Highly variable genes were used to account for the level of variance to perform a principal component analysis (PCA). When performing PCA, we aimed to solely use high-quality cells, and therefore, sources of sample-specific and cell-specific variation were regressed out. *MALAT1*, ribosomal, and mitochondrial genes were removed from the highly variable gene input (2000 most variable genes). PCA was performed using the centered and scaled highly variable gene accordingly by using the “RunPCA” function within Seurat, a selection of the number of PCs was done to account for 80% of the total variation contained in all calculated PCs (50), and the first 22 PCs were chosen. To visualize the data in a low dimension, uniform manifold approximation and projection (UMAP) was performed, using the default Seurat implementation (uwot). To account for batch effects, the Harmony version tool (v1.2.0) was applied, correcting the previously generated PCA, and a new UMAP was generated using these corrected PCs. For FindNeighbors, the nearest neighbor parameter k was set at 10. For both RunUMAP and findNeighbors functions, the dims parameter was set to 22 (PCs chosen). Clusters were then identified using the “FindClusters” function from Seurat, and the resolution parameter was set at different values (from 0.2 to 2).

**Cell type identification and annotation.**

To classify cells into cell types, we applied the automatic annotation function of the SingleR R package v2.4.0, using four reference datasets for human immune cells (BlueprintEncodeData, DatabaseImmuneCellExpressionData, HumanPrimaryCellAtlasData, and MonacoImmuneData) as guides. Cells within the same clusters, generated with the FindClusters function, were assumed to share similar expression patterns. After verifying the marker genes supporting each annotation, cell identities were assigned based on the consensus output of SingleR at a clustering resolution of 1.4. Finally, annotations were consolidated into four broader categories: naïve, memory, regulatory (Tregs), and activated T cells.

**T cell receptor annotation**

Clonotype information was extracted from the Seurat object metadata and analyzed to identify shared TCR β-chain sequences across samples. Clonotype sequences were exported in FASTA format and merged with curated TCR sequences from the VDJdb database. To group similar TCRs, clustering was performed with CD-HIT v4.8.1 at 85% sequence identity, and the resulting cluster metadata were parsed and reintegrated into the Seurat object. Each cell was then annotated according to its TCR cluster and corresponding disease associations reported in VDJdb, including SARS-CoV-2, CMV, EBV, autoantigens, and other pathogens, based on cluster annotation pattern matching.

**Differential gene expression.**

The differential expression analysis was performed using a pseudobulk approach. Single-cell RNA-seq data were aggregated into pseudobulk profiles by summing expression counts for each gene across cells of the same cell type and individual, using an in-house script based on the Libra R package. The to_pseudobulk() function of Libra was modified and used to filter individuals with a minimum number of cells (10 cells), for each cell type comparison. Differential expression was conducted using DESeq2, with a Wald test for statistical significance. Significant genes were identified based on an adjusted p-value threshold (False Discovery Rate (FDR - padj) < 0.1).

Gene set enrichment analysis (GSEA) was applied using the package fgsea v1.30.0. We used the pre-ranked mode, and an ordered gene list was produced per dataset by scoring each gene according to the following formula:

$$score=-log_{10}(pvalue)\times sign(\log_{2} FC),$$

Here, *p*-value from the differential expression analysis for each gene, and log_2_FC denotes the corresponding log_2_ fold-change value derived from comparisons between groups. Similarities in gene dysregulation patterns between groups were evaluated by the Normalized Enrichment Score. We used the MSigDB v2024.1.Hs gene-sets available from the Molecular Signature Database.*(7)* This database is a curated collection of 50 well-defined biological processes, which provides refined and concise inputs for gene set enrichment analysis. We removed genes not expressed in any sample from the gene sets to reduce bias.*(8)* We considered a pathway enriched if FDR <0.05.

**ELISA**

Antibody responses against SARS-CoV-2 Spike RBD, EBV EBNA1, and CMV pp65 were assessed via ELISA. Serology experiments were performed, as previously described for all plasma samples collected (n=101).*(22)* Briefly, plasma samples were thawed, heat inactivated at 56°C for 30 minutes, and centrifugated before use. Negative control plasma was pooled from healthy human donors unexposed to SARS-CoV-2 infection or vaccination. Positive control plasma, pooled from convalescent COVID, CMV, and EBV donors, was used to normalize results across separate experiments. 96-well half-area plates were coated with 1μg/mL of recombinant protein: EBNA1(EBV - Abcam ab138345); pp65(CMV - Abcam ab43041, SARS-CoV-2 Spike RBD (Donated from Prof. Saphire Lab, LJI) and SARS-CoV-2 Nucleocapsid (GenScript - Z03488). Recombinant protein was diluted in PBS and incubated overnight at 4°C. After washing, plates were blocked for 90 minutes with 3% milk and 0.05% Tween-20 in PBS. Heat-inactivated plasma samples were diluted in 1% milk and 0.05% Tween-20 PBS, serially diluted, and incubated for 90 minutes. Plates were washed five times with 0.5% Tween-20 in PBS, followed by a 60-minute incubation with the conjugated secondary antibody. After washing, plates were developed with TMB substrate and read at 450 nm. The limit of sensitivity (LOS) was determined from negative control plasma. The limit of detection (LOD) was defined as 1:3. The difference between antibody titers was evaluated using the Wilcoxon rank sum test.

**Neutralization assay**

The SARS-CoV-2 pseudovirus (PSV) neutralization assay was performed for timepoint 3 samples as previously described.*(22)* Briefly, a monolayer of Vero cells (ATCC, Cat# CCL-81) was generated by seeding 2.5x10^4^ cells in flat clear-bottom black 96-well plates (Corning, Cat# 3904). Recombinant SARS-CoV-2-spike pseudotyped VSV-ΔG-GFP was generated with the specific amino acid mutation D614G (WT). Pre-titrated recombinant viruses for each variant were incubated with serially diluted human heat-inactivated plasma at 37°C for 1-1.5 hours. Confluent Vero cell monolayers were added and incubated for 16 hours at 37°C in 5% CO_2_, then fixed in 4% paraformaldehyde in PBS pH 7.4 (Santa Cruz, Cat# sc-281692) with 10 μg/ml Hoechst (Thermo Scientific, Cat#62249). Cells were imaged using a Cell Insight CX5 imager to quantify the total number of cells and infected GFP-expressing cells to determine the percentage of infection. Neutralization titers (inhibition dose 50-ID50) were calculated using the One-Site Fit Log IC50 model in Prism 8.0 (GraphPad). The difference between neutralization titers was evaluated using the Wilcoxon rank sum test.

# Supplementary Table 1. Log_2_ Fold changes and p-values of differentially expressed genes in the comparison *activated__AIM+_*

| **Gene** | **log_2_FoldChange** | **p-value** | **False discovery rate** |
| --- | --- | --- | --- |
| GEM | 5.94 | 2.04E-04 | 0.0958 |
| ANK3 | -2.34 | 1.68E-05 | 0.0217 |
| NR4A1 | 2.30 | 4.35E-05 | 0.0449 |
| CD38 | 1.99 | 9.75E-05 | 0.0629 |
| GNG4 | 1.87 | 8.37E-05 | 0.0617 |
| CST7 | 1.76 | 2.25E-04 | 0.0967 |
| RGCC | 1.72 | 9.67E-07 | 0.0025 |
| SRGN | 1.28 | 3.21E-11 | 0.0000 |
| GPR183 | -1.27 | 1.57E-04 | 0.0841 |
| IFITM1 | 1.09 | 5.90E-05 | 0.0507 |
| GNAS | 1.09 | 1.63E-04 | 0.0841 |
| CD7 | 1.07 | 3.21E-06 | 0.0055 |

# Supplementary Table 2. Log_2_ Fold changes and p-values of differentially expressed genes in the comparison *Treg__AIM+_*

| **Gene** | **log_2_FoldChange** | **p-value** | **False discovery rate** |
| --- | --- | --- | --- |
| GSTP1 | 2.55 | 1.48E-05 | 0.0043 |
| LGALS1 | 2.49 | 1.57E-03 | 0.0930 |
| TXN | 2.37 | 8.89E-10 | 0.0000 |
| LSP1 | 1.95 | 8.32E-04 | 0.0803 |
| TNFRSF4 | 1.89 | 7.68E-10 | 0.0000 |
| CNBP | 1.82 | 1.20E-03 | 0.0912 |
| UBALD2 | 1.66 | 1.60E-03 | 0.0930 |
| RBM17 | 1.57 | 6.14E-04 | 0.0666 |
| RPS26 | 1.42 | 3.09E-04 | 0.0447 |
| CYBA | 1.40 | 2.31E-05 | 0.0050 |
| RPS9 | 1.26 | 1.27E-04 | 0.0220 |
| CD3D | 1.21 | 1.26E-03 | 0.0912 |
| FTL | 1.18 | 1.16E-03 | 0.0912 |
| PFN1 | 0.97 | 1.60E-03 | 0.0930 |
| IL32 | 0.94 | 3.91E-04 | 0.0486 |

# Supplementary Table 3. Log_2_ Fold changes and p-values of differentially expressed genes in the comparison *Naïve__Naïve_*

| **Gene** | **log_2_FoldChange** | **p-value** | **False discovery rate** |
| --- | --- | --- | --- |
| PCDH11X | -4.19 | 5.49E-05 | 0.0962 |
| TIGIT | -2.88 | 7.59E-05 | 0.0977 |
| POLR2J2 | -2.55 | 2.30E-05 | 0.0484 |
| MIR155HG | -2.34 | 9.36E-05 | 0.0983 |
| ANXA1 | -1.65 | 1.49E-05 | 0.0484 |
| GPR183 | -1.44 | 8.37E-05 | 0.0977 |
| MT-ATP8 | -1.41 | 1.90E-05 | 0.0484 |
| CD99 | -1.21 | 8.10E-06 | 0.0425 |
| TPP1 | -0.83 | 7.55E-05 | 0.0977 |
| CD7 | -0.83 | 1.90E-06 | 0.0200 |


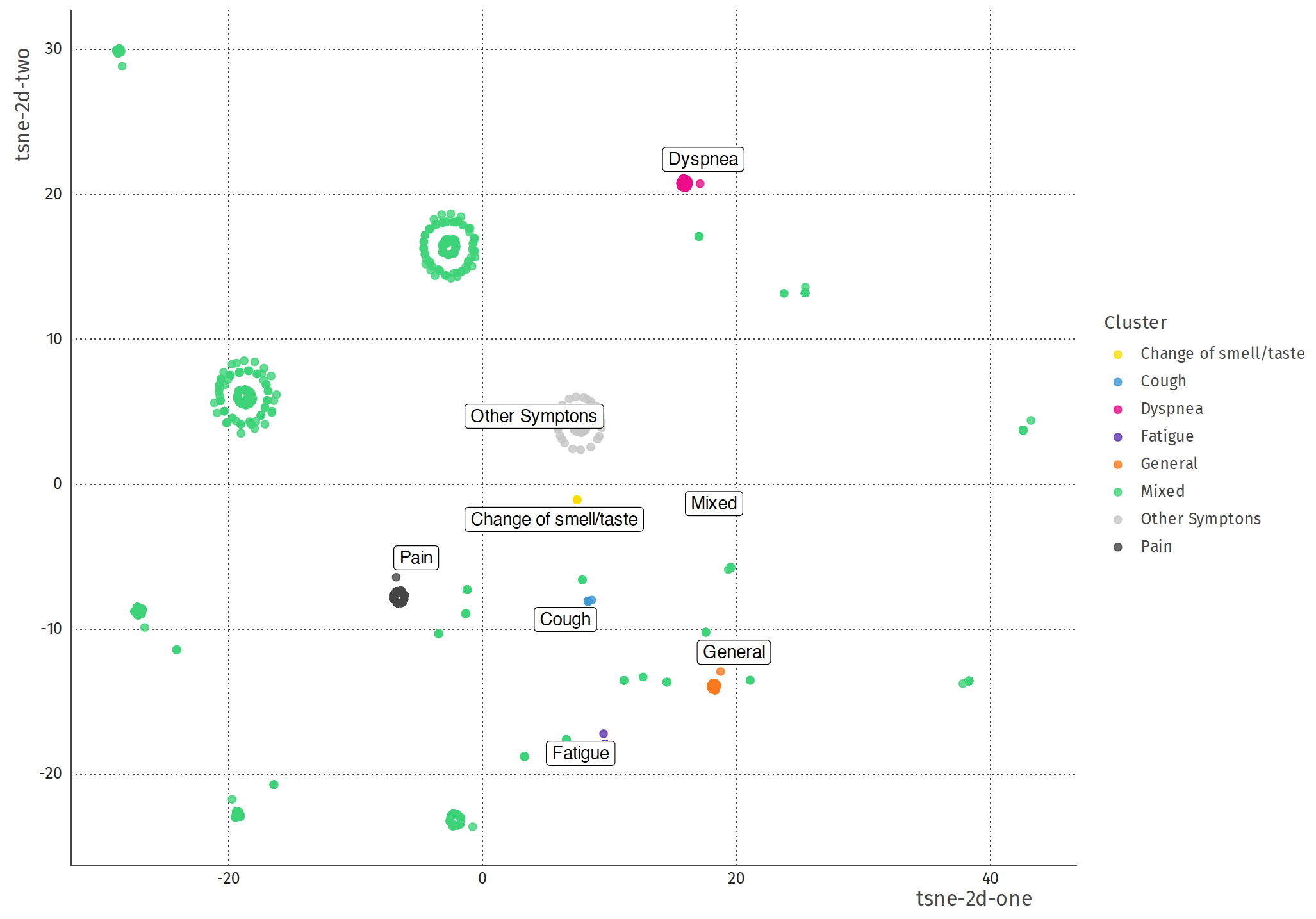


# Supplementary Figure 1: Dimensionality reduction through t-Distributed Stochastic Neighbor Embedding (t-SNE). Evaluating the presence of cough, fatigue, dyspnea, pain symptoms (headache, thoracic and muscle pain), and changes in taste and smell.


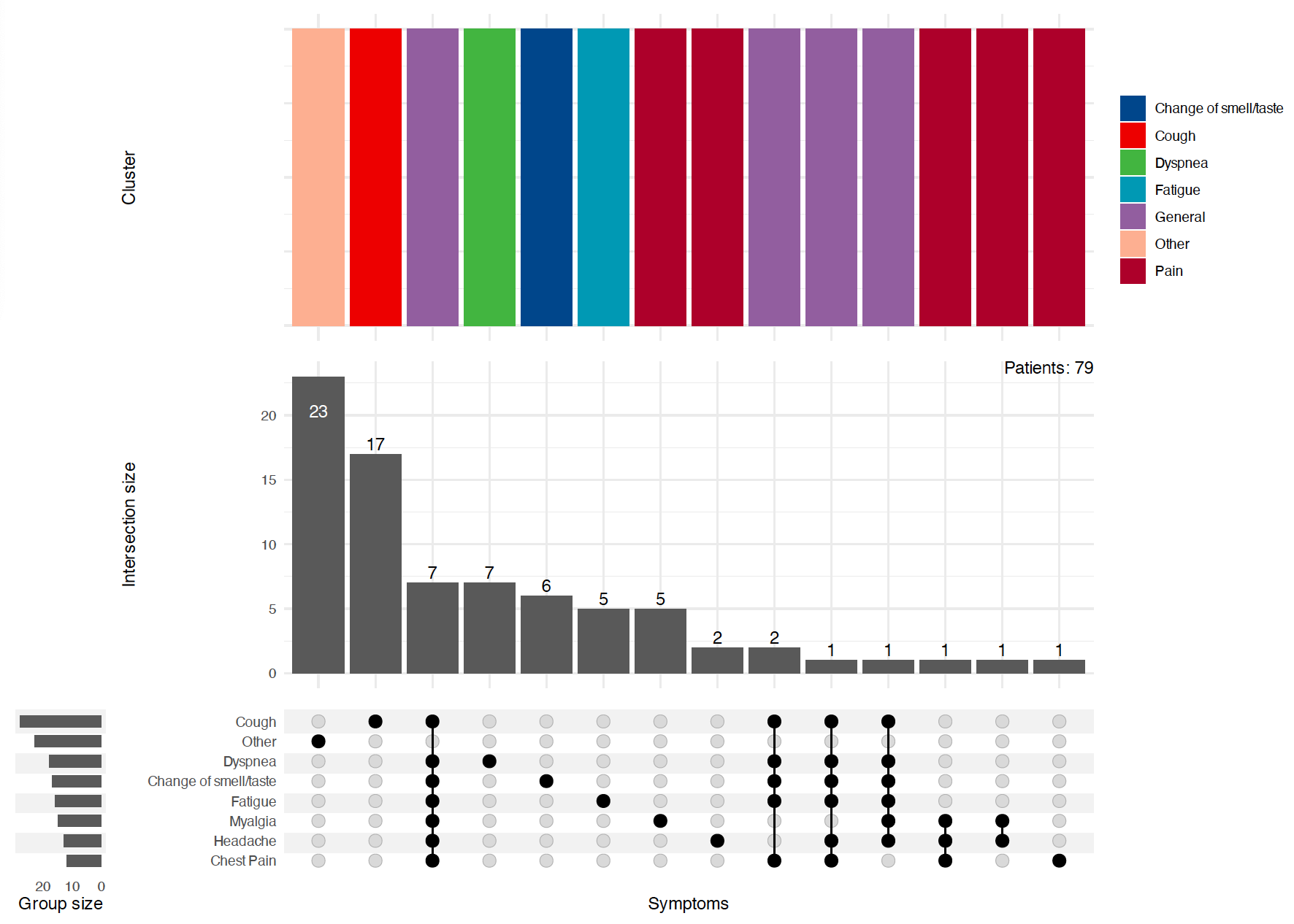


# Supplementary Figure 2: Number of participants with each combination of symptoms and related cluster.


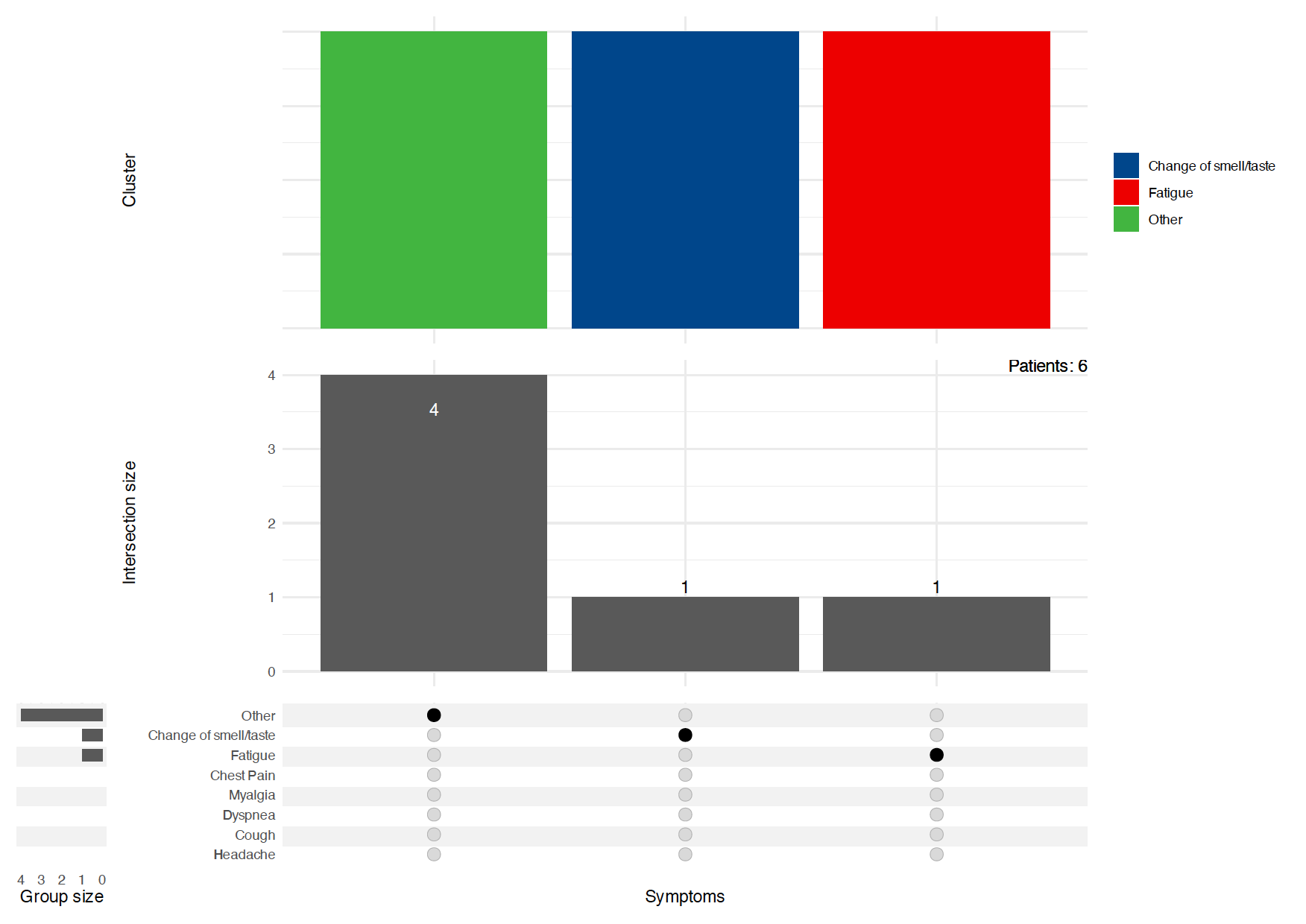


# Supplementary Figure 3: Number of participants with each combination of symptoms and related cluster for the T-cell study.


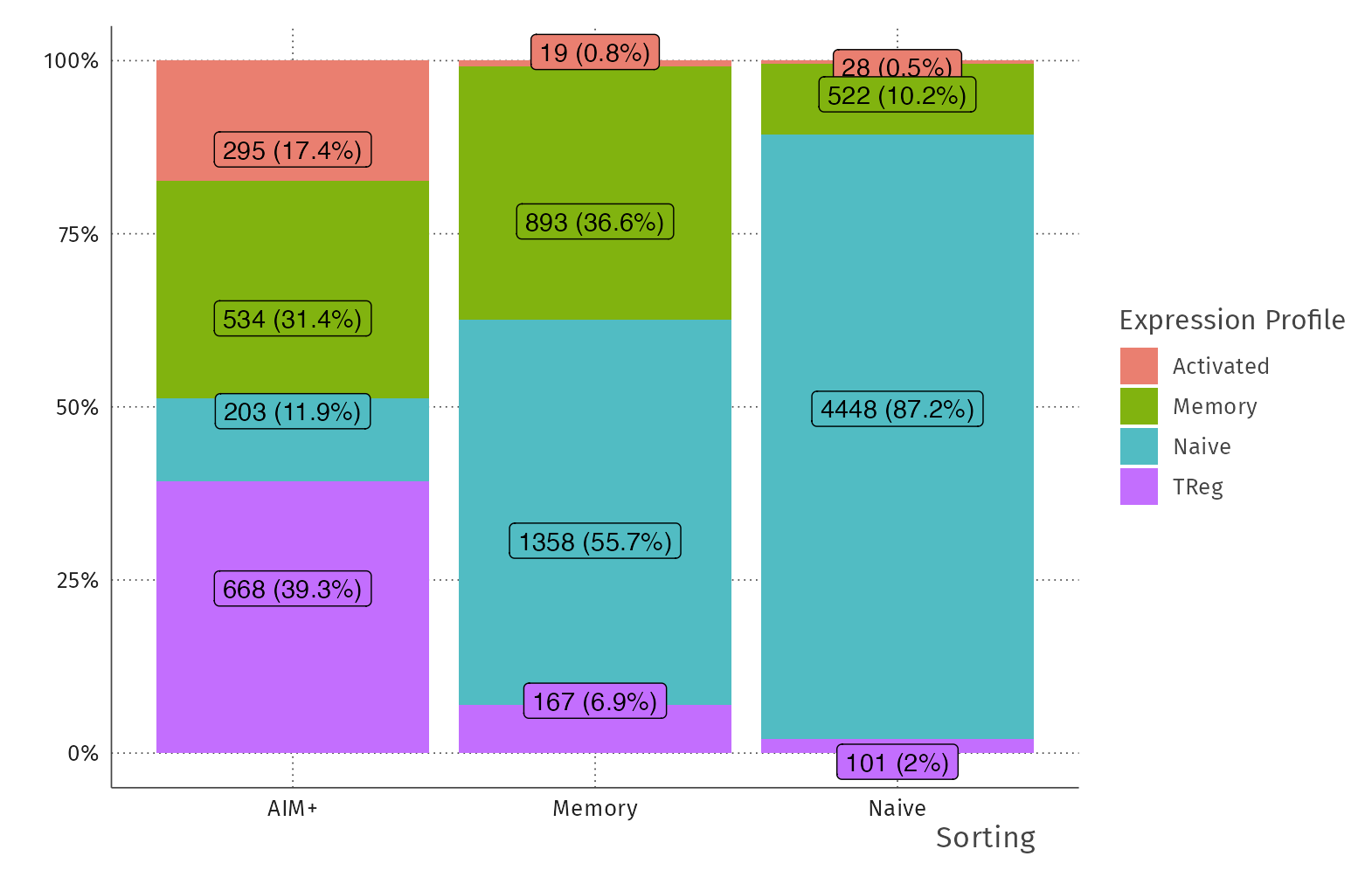


# Supplementary Figure 4: Percentage of cells by gene expression profile (scRNA-seq) within each sorting (flow cytometry)


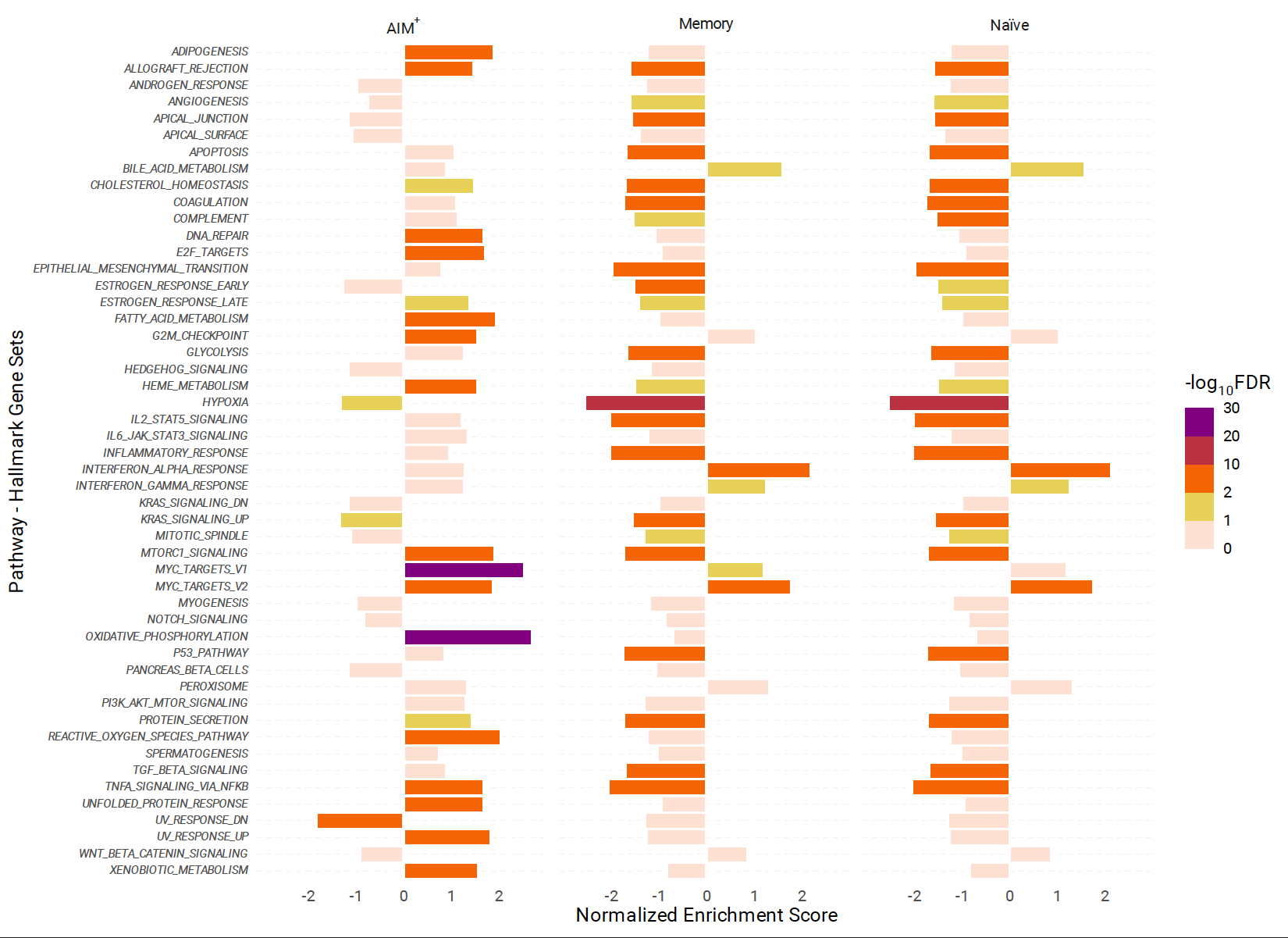


# Supplementary Figure 5: GSEA by cell sorting. A. AIM^+^, B. Memory, C. Naïve


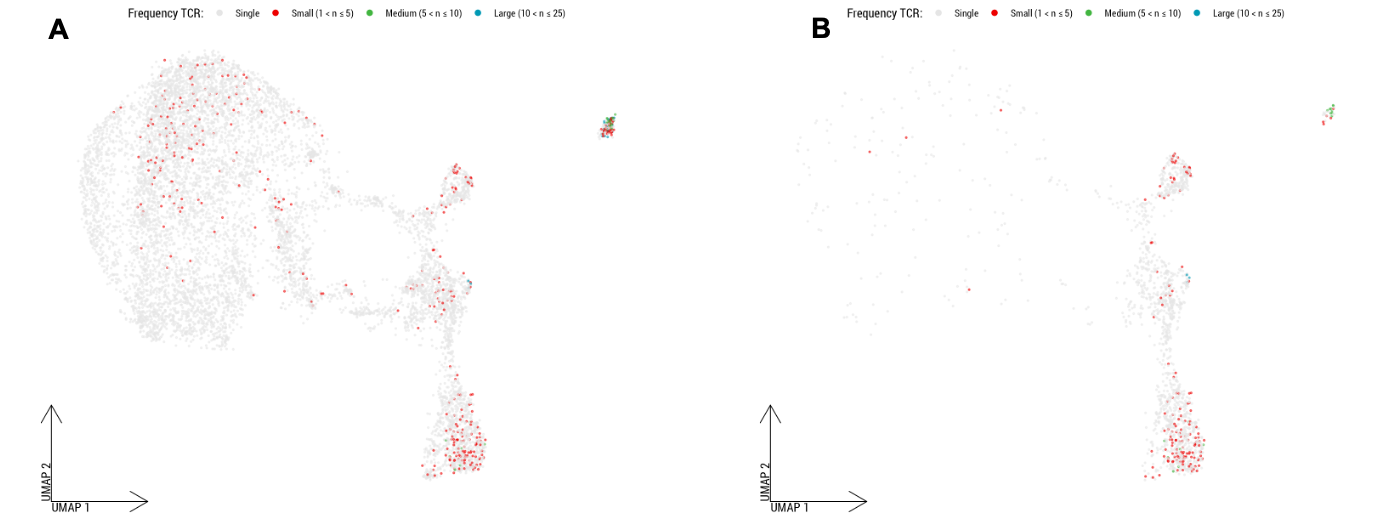


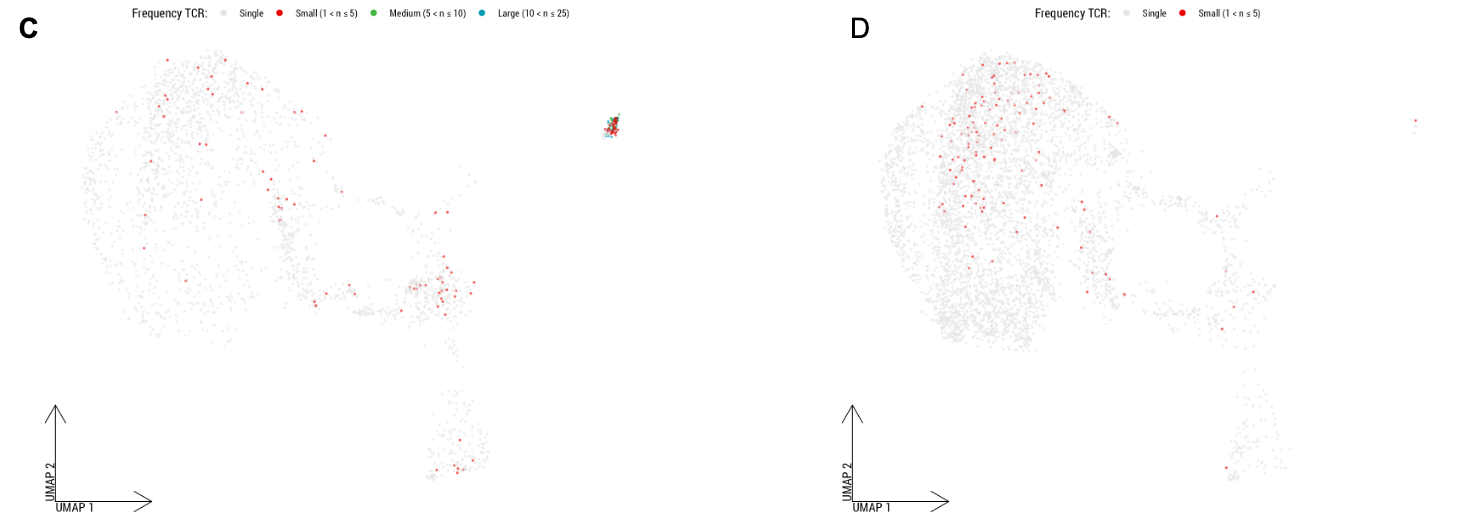


# Supplementary Figure 6: TCR Expansion overall and by cell sorting. A. Overall, B. AIM+, C. Memory, D. Naïve

# Supplementary References

1. Barreto APA, Filho MAB, Duarte LC, Cerqueira-Silva T, Camelier A, Tavares NM, et al. Metabolic disorders and post-acute hospitalization in black/mixed-race patients with long COVID in Brazil: A cross-sectional analysis. PLOS ONE. 2022 Oct 31;17(10):e0276771.

2. Wanga V. Long-Term Symptoms Among Adults Tested for SARS-CoV-2 — United States, January 2020–April 2021. MMWR Morb Mortal Wkly Rep [Internet]. 2021 [cited 2025 June 11];70. Available from: https://www.cdc.gov/mmwr/volumes/70/wr/mm7036a1.htm

3. D’Orazio M. Gower’s similarity coefficients with automatic weight selection [Internet]. arXiv; 2024 [cited 2025 June 11]. Available from: http://arxiv.org/abs/2401.17041

4. Van der Laan M, Pollard ,Katherine, and Bryan J. A new partitioning around medoids algorithm. Journal of Statistical Computation and Simulation. 2003 Aug 1;73(8):575–84.

5. Havenar-Daughton C, Reiss SM, Carnathan DG, Wu JE, Kendric K, Torrents de la Peña A, et al. Cytokine-Independent Detection of Antigen-Specific Germinal Center T Follicular Helper Cells in Immunized Nonhuman Primates Using a Live Cell Activation-Induced Marker Technique. J Immunol. 2016 Aug 1;197(3):994–1002.

6. Zheng GXY, Terry JM, Belgrader P, Ryvkin P, Bent ZW, Wilson R, et al. Massively parallel digital transcriptional profiling of single cells. Nat Commun. 2017 Jan 16;8(1):14049.

7. Liberzon A, Subramanian A, Pinchback R, Thorvaldsdóttir H, Tamayo P, Mesirov JP. Molecular signatures database (MSigDB) 3.0. Bioinformatics. 2011 June 15;27(12):1739–40.

8. Timmons JA, Szkop KJ, Gallagher IJ. Multiple sources of bias confound functional enrichment analysis of global -omics data. Genome Biology. 2015 Sept 7;16(1):186.
